# Balance Control in Children and Youth with Autism Spectrum Disorder: A Systematic Review and Meta-Analysis

**DOI:** 10.31083/AP42869

**Published:** 2025-06-04

**Authors:** Wenhong Xu, Niuniu Li, Jing Qi

**Affiliations:** ^1^College of Physical Education and Health Sciences, Zhejiang Normal University, 321004 Jinhua, Zhejiang, China

**Keywords:** children and adolescents, autism, balance, systematic review, meta-analysis

## Abstract

**Background:**

Compared with typically developing (TD) children and youth, those with autism spectrum disorder (ASD) present more balance deficits. However, the understanding of which specific balance areas are affected remains incomplete at present.

**Methods:**

Relevant studies were searched in PubMed, Web of Science Core Collection, Scopus and EBSCO from the establishment of the database to March 17, 2024. Two reviewers independently screened the literature, extracted data and assessed the methodological quality of the included studies. Meta-analysis was performed through Review Manager software, and a narrative description of the results was used if the data could not be pooled for meta-analysis.

**Results:**

A total of 16 studies were included, with six being suitable for meta-analysis. The research indicated that individuals with ASD showed poorer balance control compared with TD peers. Specifically, the ASD group faced significant difficulties in sensory orientation and demonstrated deficiencies in verticality and anticipatory postural adjustments.

**Conclusions:**

Children and youth with ASD exhibit impairments in balance control across different domains compared with their TD peers. More research is needed to comprehensively assess the balance control construct in this population, including studies with longitudinal designs in particular.

**The PROSPERO Registration:**

The protocol of this systematic review was registered with the International Prospective Register of Systematic Reviews (registration no. CRD42024553855; registration date 15 June 2024; https://www.crd.york.ac.uk/PROSPERO/view/CRD42024553855).

## Main Points

1. This review provides an overall understanding of balance deficits in children and youth with autism spectrum disorder (ASD) by contrasting balance performance with that of their typically developing (TD) peers.

2. Children and youth with ASD presented more poorly within different balance control areas than their TD peers.

3. Further studies are warranted to assess the entire construct of balance control among individuals with ASD, with longitudinal designs encouraged.

## 1. Introduction

Autism spectrum disorder (ASD) is a neurodevelopmental disorder characterised by restricted repetitive behaviours, impaired communication and limited social interaction [1]. Globally, ASD has a prevalence of approximately 1% amongst children and its estimated prevalence has increased over time [2]. Children with ASD frequently present with health-related problems, including gastrointestinal issues, epilepsy, sleep–wake disturbances, immune abnormalities and chronic diseases, for example, obesity and diabetes [3, 4, 5]. Motor difficulties, such as delayed motor development, decreased muscle strength, joint hypermobility and postural deficits, are also common in this population [6, 7, 8]. In children with ASD, impaired ability of postural control during infancy leads to delayed motor development, such as crawling and walking [9]. Subsequently, when ASD children walk upright, the gravitational effect and decreased postural stability manifest as balance control deficiencies [9].

Balance control, also known as postural control, is the ability to maintain or control the centre of gravity within a base of support to prevent falls and complete desired movements [10]. This capability can be examined under either static (the body remains motionless) or dynamic (the body reacts to an external perturbation or is in motion) conditions, as well as under both conditions [11]. Poor balance is a predictor of falling and always associated with low levels of self-esteem and a sedentary lifestyle, which may lead to overweight, obesity, cardiovascular disease and even increased risk of death from all causes [12]. The balance control system requires interaction amongst musculoskeletal and neural subsystems, such as vestibular, visual, auditory and proprioceptive systems [13]. Considering that deficits in balance control may result from impairment in any or all of these subsystems, the full evaluation of the various aspects of balance control through the adoption of a range of tests is needed.

The Balance Evaluation Systems Test (BESTest) is the first balance assessment tool designed to locate the impairments responsible for balance problems [13]. This conceptual framework integrates the different aspects of balance control into six domains: biomechanical constraints, stability limits and verticality, anticipatory postural adjustments, reactive postural responses, sensory orientation and gait stability. Biomechanical constraints mainly refer to balance control requisites in the musculoskeletal domain, such as ankle or hip strength, postural alignment and base of foot support. Stability limits represent how far the body’s centre of mass can move on its base of support, while verticality refers to an internal perception of gravitational uprightness. Anticipatory postural adjustments are voluntary movements of the body’s centre of mass in anticipation of a postural transition from one position to another. Reactive postural responses include both inplace and compensatory stepping responses to an external perturbation. Sensory orientation refers to the realization of spatial orientation through the integration of different sensory information. Gait stability focus on the balance during gait, which refers to the ability to catch a falling centre of mass through changing the support of foot [13, 14].

The BESTest framework aids in the identification of the various balance limitations and is important in the design of interventions focusing on identified deficits [13]. The framework has been shown to be effective during the evaluation of balance control in patients with Parkinson’s disease [15], stroke survivors [16], individuals with vestibular disorders [13], children and youth with cerebral palsy and developmental coordination disorder [14, 17]. Balance deficits have been well recognized in children and youth with ASD [9, 18]. However, a comprehensive understanding of which balance domains, or to what extent this population are affected has not been described in the literature.

Using the BESTest framework as a foundation, the aims of this systematic review and meta-analysis are to: (1) Comprehensively summarize and identify balance deficits in various domains in children and youth with ASD by comparing the balance performance of this population with that of their typically developing (TD) peers and (2) Provide recommendations for future research. The findings of this review should assist physical therapists, PE teachers and policy makers identify which balance domains are impaired in children and youth with ASD and the development of pertinent interventions to improve their balance control abilities. Targeted balance improvements enhance the overall motor proficiency of individuals with ASD, which in turn can have a positive impact on their social functioning. The perspective and outcomes provided by this review therefore not only contributes to a comprehensive understanding of balance control in children and youth with ASD, but also offers insights for future research and clinical practice aimed at improving the social function of this population.

## 2. Methods

The Preferred Reporting Items for Systematic Reviews and Meta-Analysis (PRISMA) guidelines were followed to search literature, select studies and extract information [19]. The PRISMA 2020 checklist is included in the **Supplementary Material-PRISMA_2020_checklist**. The protocol of this systematic review was registered with the International Prospective Register of Systematic Reviews (registration no. CRD42024553855; registration date June 15, 2024; https://www.crd.york.ac.uk/PROSPERO/).

### 2.1 Information Sources and Search Strategy

PubMed, Web of Science Core Collection, Scopus and EBSCO were systematically searched on March 17, 2024, without filter application. Wenhong Xu, Niuniu Li and Jing Qi agreed on the search strategy, which covered the following search subject areas: (1) Autism, (2) Balance and (3) Children or youth. **Supplementary File 1** provides additional details about the search strategy.

### 2.2 Selection Criteria

Relevant studies were identified in accordance with the following selection criteria based on the Population Intervention Comparison Outcome Study design method [20].

**Population**: Individuals between 5–18 years old [21], diagnosed with ASD according to the fifth edition of the Diagnostic and Statistical Manual of Mental Disorders. As the majority of children and youth on the autism spectrum have one or more co-occurring health conditions (e.g., intellectual disabilities, attention-deficit hyperactivity disorder and anxiety disorder), ASD participants with and without comorbidities were included.

**Comparison**: One or more control groups were necessary for comparison. The performance of children and youth with ASD in balance assessment was compared to that of TD peers.

**Outcome**: Balance control of participants was measured via a standardised assessment tool (e.g., specific balance tests or balance subscales of a generic development motor scale) and reported in detail.

**Study design and publication type**: Studies with case-control designs were included in this study. Additionally, only articles that were published in English, refereed in journals with full-text availability and contained only original research (i.e., reviews were excluded) were included.

### 2.3 Literature Screening and Data Extraction

Two reviewers (Wenhong Xu and Niuniu Li) independently screened the literature, extracted and cross-checked the data. In case of disagreement, a third reviewer (Jing Qi) was consulted to assist in judgment. Duplicate articles were excluded, literature screening was then performed by initially reading titles and abstracts. The full text was then read to determine final inclusion. Additionally, the potential studies were identified by scanning the references of the included articles. Data extraction primarily included the following information: (1) Studies details (e.g., first author, the publication year, geographic location and study design), (2) Participant characteristics (e.g., sample size, age range, percentage of males and severity of ASD), (3) Key elements of risk of bias assessment and (4) Tests/tasks applied to assess participants’ balance performance and outcome indicators.

The numeric values (mean and standard deviation) of outcome variables were extracted. The data from a specific balance test were pooled and grouped into the corresponding domains of balance control. The values of the data derived from a balance scale or subscale of a generic motor scale were also extracted and analysed.

### 2.4 Risk of Bias Assessment

The Scottish Intercollegiate Guidelines Network (SIGN) checklist was used to assess the methodological quality of the included studies [22]. The SIGN checklist comprises two sections. Section 1 involves an evaluation of the internal validity of a study and includes 11 items: Research question (one item), sampling (six items), measurement (two items), confounding control (one item) and data analysis (one item). Section 2 provides an overall assessment of each study via the classification of three degrees. When most criteria were met (≥9/11) or (6–8/11), a study was rated to be of high (++) or acceptable (+) quality. A study was classified as low quality (0) when criteria were not met (≤5/11) or substantial flaws existed in key aspects related to study design. The first and second authors independently performed methodological assessment. Cohen’s kappa analysis was applied to measure the level of consistency amongst authors: Poor (≤0), slight (0.0–0.20), fair (0.21–0.40), moderate (0.41–0.60), substantial (0.61–0.80) and almost perfect (0.81–1.0) [23]. Studies rated to have low methodological quality on the basis of the SIGN checklist were excluded [22].

### 2.5 Statistical Analysis and Synthesis

The included studies revealed various balance deficiencies in children and youth with ASD. A meta-analysis was conducted if at least two identical result variables for the same test were available to comprehensively summarise the evidence in the current literature. Review Manager (Version 5.4.1, The Cochrane Collaboration, Copenhagen, Denmark) was used for data processing. Standardised mean difference (SMD) with a 95 % confidence interval (CI) was applied to analyse effect size. When statistical heterogeneity was found across studies (*I*^2^ ≥ 50 %, *p* < 0.10), a random-effects model was applied; otherwise, a fixed-effects model was used. Hedges’ *g* method was employed to reflect the magnitude of effect size [24]. According to Cohen (1988) [25], small, medium and large effect sizes have values between ≥0.2 and <0.5, between ≥0.5 and <0.8 and ≥0.8. When a study contained two or more control groups, each result was analysed separately. Statistical significance was set at *p* < 0.05 for the comparisons between groups. Sensitivity analysis was conducted to test if the results of the meta-analysis are robust. Funnel plot was made by Review Manager 5.4.1 software, and sensitivity analysis and Egger’s test were analysed by Stata 18.0 (Stata Corporation, College Station, TX, USA). For inappropriate data or those that were impossible to pool quantitatively, descriptive analysis was conducted and a narrative summary was provided.

## 3. Results

### 3.1 Search Results

**Fig. 1.**
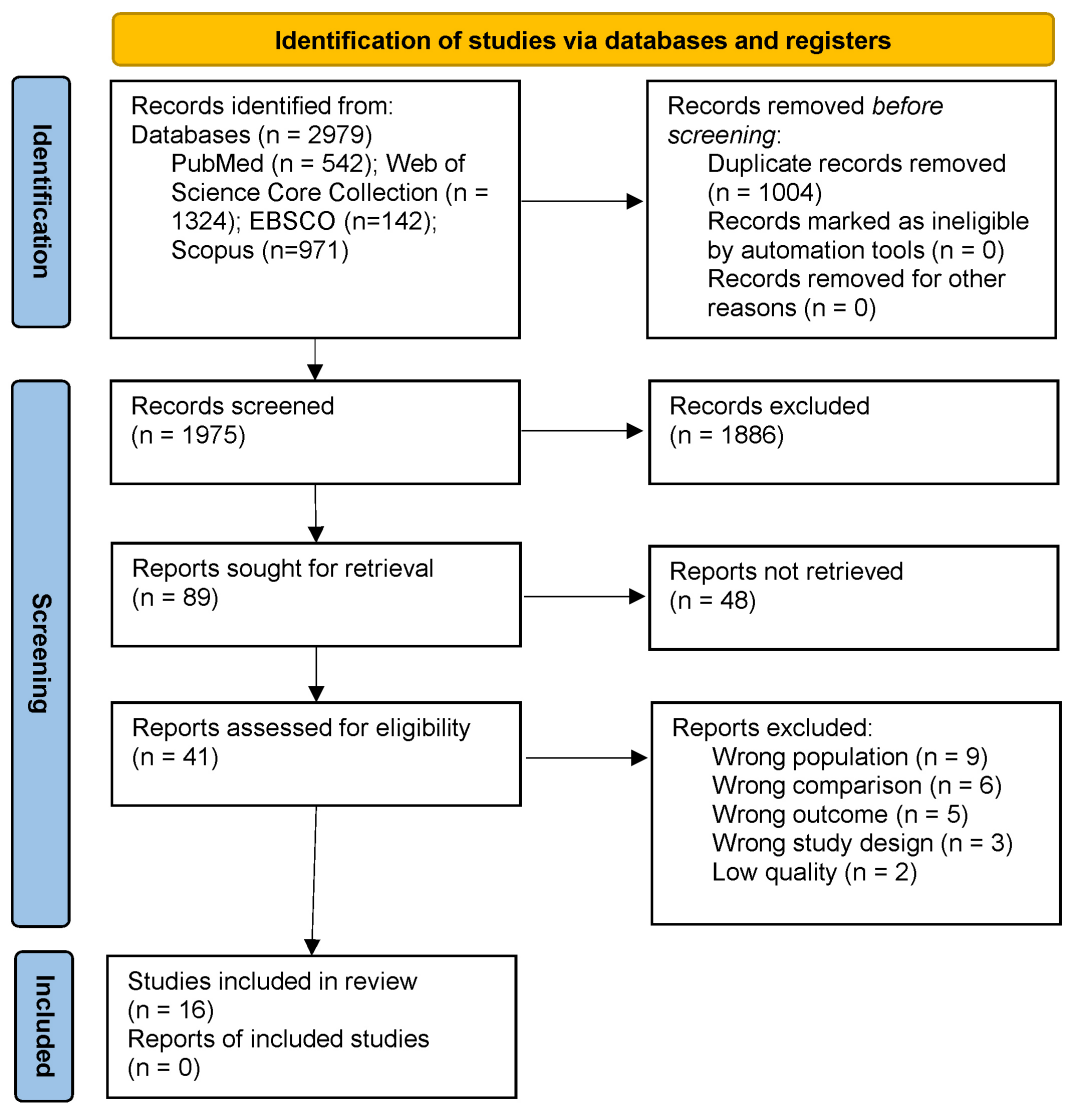
**PRISMA flow diagram for the identification, screening, eligibility and inclusion of studies**.

The initial search identified 2979 studies. These studies were exported to EndNote X9 (Clarivate Analytics, Philadelphia, PA, USA) and duplicates were eliminated. The remaining 1975 articles were then screened based on their titles and abstracts. This step resulted in the exclusion of 1886 studies. The remaining 89 articles were reviewed in full text and another 71 articles excluded. Amongst the 18 studies that met the selection criteria, two were excluded due to their low methodological quality. Therefore, 16 studies were ultimately identified for this review. The PRISMA flowchart in Fig. 1 depicts the selection procedure.

### 3.2 Risk of Bias Assessment

Table 1 (Ref. [9, 18, 26, 27, 28, 29, 30, 31, 32, 33, 34, 35, 36, 37, 38, 39, 40, 41]) outlines the assessment results for methodological quality. Overall, out of the 18 studies, one was rated as high quality [26], 15 were of acceptable quality [9, 18, 27, 28, 29, 30, 31, 32, 33, 34, 35, 36, 37, 38, 39] and two had low quality [40, 41]. The methodological quality assessment indicated an ‘almost perfect’ inter-rater agreement for these studies (kappa value: 0.817, standard error: 0.038, 95 % CI: 0.743–0.892) [23].

**Table 1.** **Risk of bias assessment of individual studies**.

Study	1	2	3	4	5	6	7	8	9	10	11	Overall assessment
Kohen-Raz *et al*. 1992 [32]	+	+	–	NR	–	+	+	–	+	+	–	A
Molloy *et al*. 2003 [38]	+	+	–	ASD: 25%	–	+	+	?	+	+	–	A
				TD: NR								
Minshew *et al*. 2004 [37]	+	+	+	NR	–	+	+	?	+	+	–	A
Fournier *et al*. 2010 [30]	+	+	+	NR	–	+	+	?	+	+	–	A
Liu & Breslin, 2013 [35]	+	+	+	NR	–	+	+	+	+	+	–	A
Memari *et al*. 2013 [36]	+	+	+	NR	–	+	+	?	+	+	+	A
Fournier *et al*. 2014 [31]	+	+	+	NR	–	+	+	?	+	+	–	A
Ament *et al*. 2015 [28]	+	+	+	NR	–	+	+	?	–	+	+	A
Stins *et al*. 2015 [41]	+	+	?	NR	–	+	+	?	–	+	–	L
Wang *et al*. 2016 [39]	+	+	+	NR	–	+	+	?	+	+	–	A
Lidstone *et al*. 2020 [33]	+	?	+	NR	–	+	+	?	+	+	–	A
Lim *et al*. 2020 [34]	+	+	–	NR	–	+	+	?	+	+	+	A
Lourenço *et al*. 2020 [40]	+	+	+	NR	–	+	+	?	–	–	–	L
Hu *et al*. 2021 [26]	+	+	+	ASD: 82.9%	+	+	+	?	–	+	+	H
				TD: NR								
Abdel Ghafar *et al*. 2022 [27]	+	?	?	ASD: 92.7%	–	+	+	?	+	+	–	A
				TD: NR								
Faber *et al*. 2022 [29]	+	+	+	NR	–	+	+	?	+	+	–	A
Odeh *et al*. 2020 [9]	+	+	+	NR	–	+	+	?	+	+	–	A
Stania *et al*. 2023 [18]	+	?	?	NR	–	+	+	?	+	+	+	A

Note: 1: the study addresses an appropriate and clearly focused question; 2: the cases and controls are taken from comparable populations; 3: the same exclusion criteria are used for both cases and controls; 4: what percentage (%) of each group (cases and controls) participated in the study; 5: comparison is made between participants and non-participants to establish their similarities or differences; 6: cases are clearly defined and differentiated from controls; 7: it is clearly established that controls are non-cases; 8: measures will have been taken to prevent knowledge of primary exposure influencing case ascertainment; 9: exposure status is measured in a standard, valid and reliable way; 10: the main potential confounders are identified and taken into account in the design and analysis; 11: confidence intervals are provided. “+”: yes, the study does this; “–”: no, the study does not do this; “?”: can’t say whether the study does this. Abbreviations: ASD, autism spectrum disorder; TD, typically developing; H, high quality; A, acceptable quality; L, low quality; NR, not reported.

### 3.3 Study Characteristics

Table 2 (Ref. [9, 18, 26, 27, 28, 29, 30, 31, 32, 33, 34, 35, 36, 37, 38, 39]) presents the study characteristics. The included studies were published between 1992 and 2023 and more than half were conducted in the USA (*n* = 9, 56.3%). All research included, with the exception of one longitudinal study, employed a cross-sectional design. Balance control was assessed in 604 participants with ASD and 714 TD participants. The majority of studies reported mean age (ASD group: 11.37 ± 5.46 years; TD group: 11.02 ± 4.81 years) and gender distribution (ASD group: 82 % male; TD group: 79 % male).

**Table 2.** **Main characteristics of the included studies**.

Study	Country	Design	Sample size ASD	% Males ASD	Age ASD	ASD Type	Samples size TD	% Males TD	Age TD
Kohen-Raz *et al*. 1992 [32]	Israel	CS	91	71.4%	6–20	ASD	166	NR	4–11
Molloy *et al*. 2003 [38]	USA	CC	8	100%	5–12	ASD	8	100%	5–12
Minshew *et al*. 2004 [37]	USA	CS	79	89.9%	17.0 ± 10.4	HFA	61	90.2%	16.7 ± 10.5
Fournier *et al*. 2010 [30]	USA	CS	13	NR	11.1 ± 2.3	ASD	12	NR	12.9 ± 2.1
Liu & Breslin, 2013 [35]	USA	CS	30	83.3%	3–16	ASD	30	53.3%	3–16
Memari *et al*. 2013 [36]	Iran	CS	21	100%	11.5 ± 1.6	HFA	30	100%	11.6 ± 1.9
Fournier *et al*. 2014 [31]	USA	CS	16	NR	5.5 ± 1.1	ASD	17	NR	6.2 ± 1.2
Ament *et al*. 2015 [28]	USA	CS	56	85.7%	10.27 ± 1.28	ASD	81	85.2%	10.31 ± 1.18
Wang *et al*. 2016 [39]	USA	CS	22	86.4%	12.72 ± 3.64	ASD	21	85.7%	11.67 ± 4.53
Lidstone *et al*. 2020 [33]	USA	CS	23	82.6%	12.4 ± 2.8	ASD	22	63.6%	11.7 ± 2.7
Lim *et al*. 2020 [34]	Australia	CS	15	80%	9.7 ± 1.3	ASD	18	66.7%	10.0 ± 1.3
Hu *et al*. 2021 [26]	China	CS	97	83.5%	8.52 ± 1.05	ASD	117	81.2%	8.47 ± 1.05
Abdel Ghafar *et al*. 2022 [27]	Saudi Arabia	CS	38	65.8%	9.57 ± 2.08	ASD	36	58.3%	10.84 ± 2.91
Faber *et al*. 2022 [29]	Netherlands	CS	67	80.6%	13.03 ± 1.12	ASD	67	80.6%	12.85 ± 1.11
Odeh *et al*. 2020 [9]	USA	CS	12	91.7%	8.71 ± 1.69	ASD	12	83.3%	8.74 ± 2.42
Stania *et al*. 2023 [18]	Poland	CC	16	68.8%	8.13 ± 1.54	ASD	16	56.3%	7.93 ± 0.88
Total			604				714		

Note: ASD, autism spectrum disorder; HFA, high-functioning autistic; TD, typically developing; CS, cross-sectional; CC, case-control; NR, not report.

### 3.4 Performance of Children and Youth with ASD on Balance Control Subscales

**Fig. 2.**
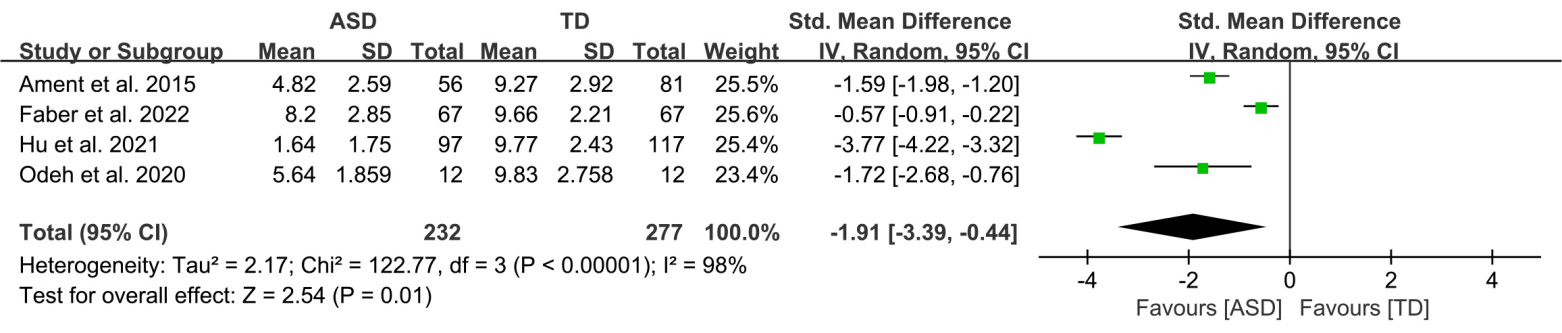
**The forest plots of the balance subscale score of the Movement Assessment Battery for Children-2 (MABC-2) meta-analysis for the autism spectrum disorder (ASD) and typically developing (TD) groups**.

Five studies, amongst which four pooled data (Fig. 2) [9, 26, 28, 29], assessed overall balance performance by using the subscale of the Movement Assessment Battery for Children-2 (MABC-2). Results showed that individuals with ASD had significantly poorer balance performance than their TD peers (SMD = –1.91, 95 % CI: –3.39 to –0.44, *p* = 0.01) but had high heterogeneity (*I*^2^ = 98 %). SMDs for the MABC-2 balance subscale were considered to have a large effect size.

One study reported the subscale score of the second edition of the Bruininks Oseretsky Test of Motor Proficiency [9]. Results also revealed that ASD group participants encountered more difficulties in balance control (SMD = –1.54, 95 % CI = –2.47 to –0.61, *p* = 0.001) than those in the TD group (**Supplementary File 2**).

### 3.5 Performance of Children and Youth with ASD on Balance Control Tests

Eleven studies adopted specific balance tests to assess balance control ability (Table 3, Ref. [18, 27, 30, 31, 32, 33, 34, 36, 37, 38, 39]). The included research involved three out of the six balanced domains, that is, sensory orientation, stability limits/verticality and anticipatory postural adjustments. Biomechanical constraints, gait stability and postural response were not covered.

**Table 3.** **The balance control domains and the applied tests/tasks for their assessment**.

Domain	Applied tests/tasks in the included studies	Applied outcome variables in the included studies
Sensory orientation	Force platform-bipedal stance-EO	COP-area [30, 31, 33, 36, 38]
		COP-AP [30, 31, 33, 36, 38]
		COP-ML [30, 31, 33, 36, 38]
		COP-AP-standard deviation [39]
		COP-ML-standard deviation [39]
		COP-length [39]
		COP-AP-length [39]
		COP-ML-length [39]
		COP-COM _max_ML_ [30]
		COP-COM _max_AP_ [30]
		COP-COM _max_R_ [30]
		COP-RMS [36]
		COP-RMS-AP [18, 33, 34, 36]
		COP-RMS-ML [18, 33, 34, 36]
		COP-RMS-AP rambling [18]
		COP-RMS-ML rambling [18]
		COP-RMS-AP trembling [18]
		COP-RMS-ML trembling [18]
		COP-V [31, 34, 36]
		COP-AP-V [33, 36]
		COP-ML-V [33, 36]
		MF [36]
		SE-AP [18, 33, 34]
		SE-ML [18, 33, 34]
		COP-AP rambling [18]
		COP-ML rambling [18]
		COP-AP trembling [18]
		COP-ML trembling [18]
	Force platform-bipedal stance-EC	COP-AP [34]
		COP-ML [34]
		COP-area [38]
		COP-RMS-AP [34]
		COP-RMS-ML [34]
		COP-V [34]
		SE-AP [34]
		SE-ML [34]
	Force platform-bipedal stance-(F) EO	COP-area [38]
	Force platform-bipedal stance-(F) EC	COP-area [38]
	Biodex balance system	Sway index [27]
		Sway index score [27]
	EquiTest	Equilibrium score [37]
Anticipatory postural adjustments	Unipedal stance test	Unipedal stance time [33]
Stability limits/verticality	Tetra-Ataxiametric Method	Stability index [32]
		Fourier spectral quotient [32]
		Weight distribution index [32]
		Toe synchronization [32]

Abbreviations: EO, eyes open; EC, eyes closed; (F) EO, (foam) eyes open; (F) EC, (foam) eyes closed; ROM, range of motion; COP, Centre of pressure; COM, Centre of mass; AP, anterior-posterior; ML, medial-lateral; V, velocity; RMS, root mean square; SE, sample entropy; MF, Mean frequency.

### 3.6 Sensory Orientation

**Fig. 3.**
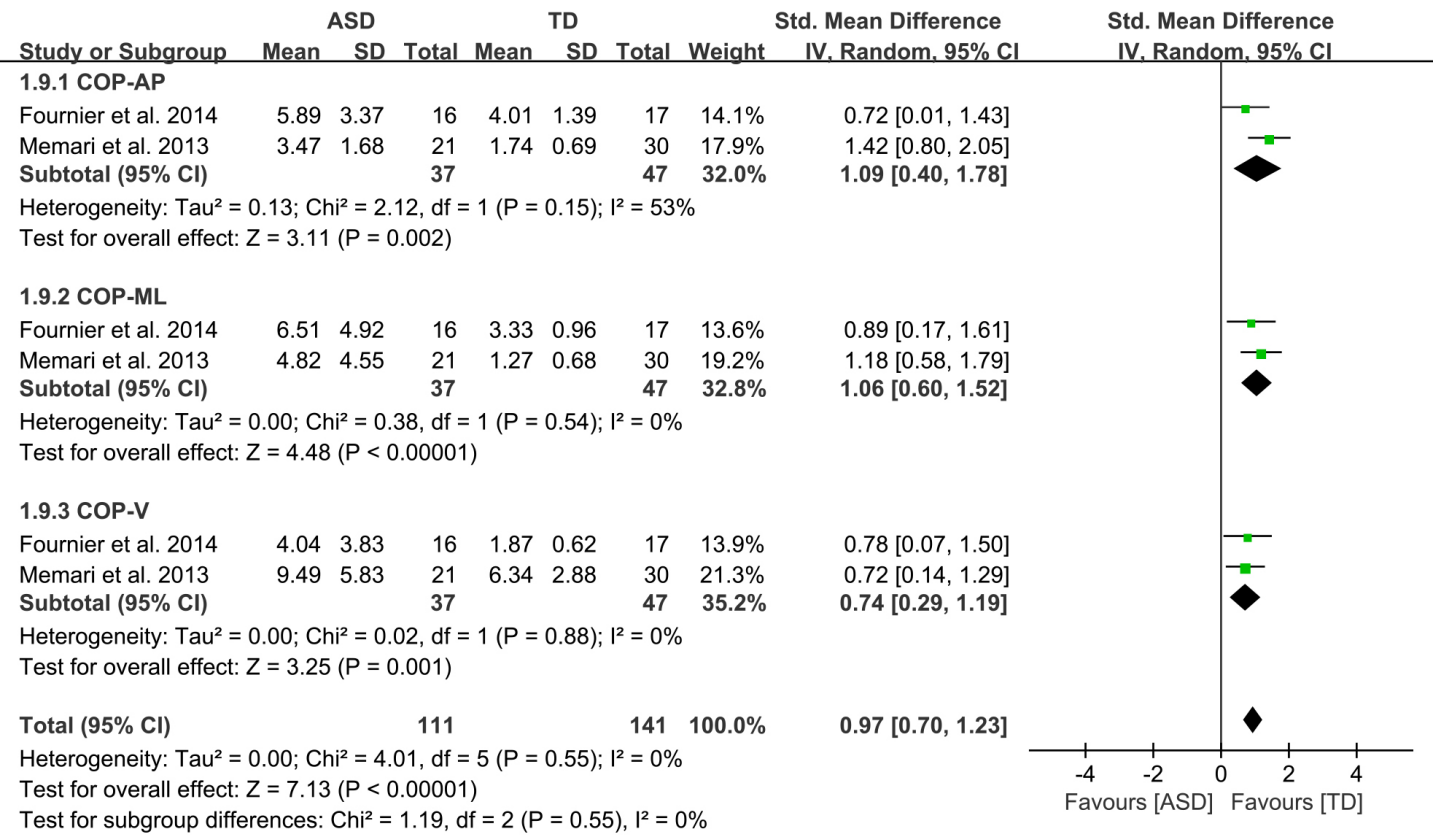
**The forest plots of force platform-standing bipedally with eyes open (sensory orientation domain) meta-analysis for the ASD and TD groups**.

Ten studies, of which two were pooled for meta-analysis, used a force platform to measure sensory orientation [31, 36]. Various parameters of the centre of pressure (COP) sway, such as velocity (V) and directions of anterior–posterior (AP) and medial–lateral (ML), were analysed. Data were pooled on the basis of the test condition of bipedal stance with eyes open (Fig. 3).

Fig. 3 shows that children and youth with ASD had a significantly higher COP sway than their TD peers during bipedal stance with eyes open (COP–AP: SMD = 1.09, 95% CI: 0.40–1.78, *I*^2^= 53%, *p* = 0.002; –ML: SMD = 1.06, 95% CI: 0.60–1.52, *I*^2^ = 0%, *p* < 0.001; –V: SMD = 0.74, 95% CI: 0.29–1.19, *I*^2^ = 0%, *p* = 0.001). The SMDs of COP sway with eyes open was considered to have a large effect size.

Although six other studies were excluded from the meta-analysis owing to differences in their outcome variables or lack of relevant data [18, 27, 30, 33, 37, 39], the extent of postural sway when participants were standing bipedally with their eyes open showed similar trends (**Supplementary File 2**). These trends were observed in other outcome variables of postural sway while standing with eyes open or closed on foam or a sway-referenced support surface (**Supplementary File 2**) [27, 37]. Additionally, two other studies indicated that the extent of postural sway in an ASD group was similar to that in a control group when the participants were standing bipedally with their eyes open [34, 38].

### 3.7 Stability Limits/Verticality

In one study, the tetra-ataxiameter method was used to assess verticality [32]. Results revealed that participants with ASD showed poorer verticality than the controls (**Supplementary File 2**). None of the included studies provided discussion on stability limits.

### 3.8 Anticipatory Postural Adjustments

A unipedal stance test was used to assess anticipatory postural adjustments in one study [33]. Results revealed that the ASD group used considerably less total time to maintain balance than the controls (**Supplementary File 2**).

### 3.9 Sensitivity Analysis

**Fig. 4.**
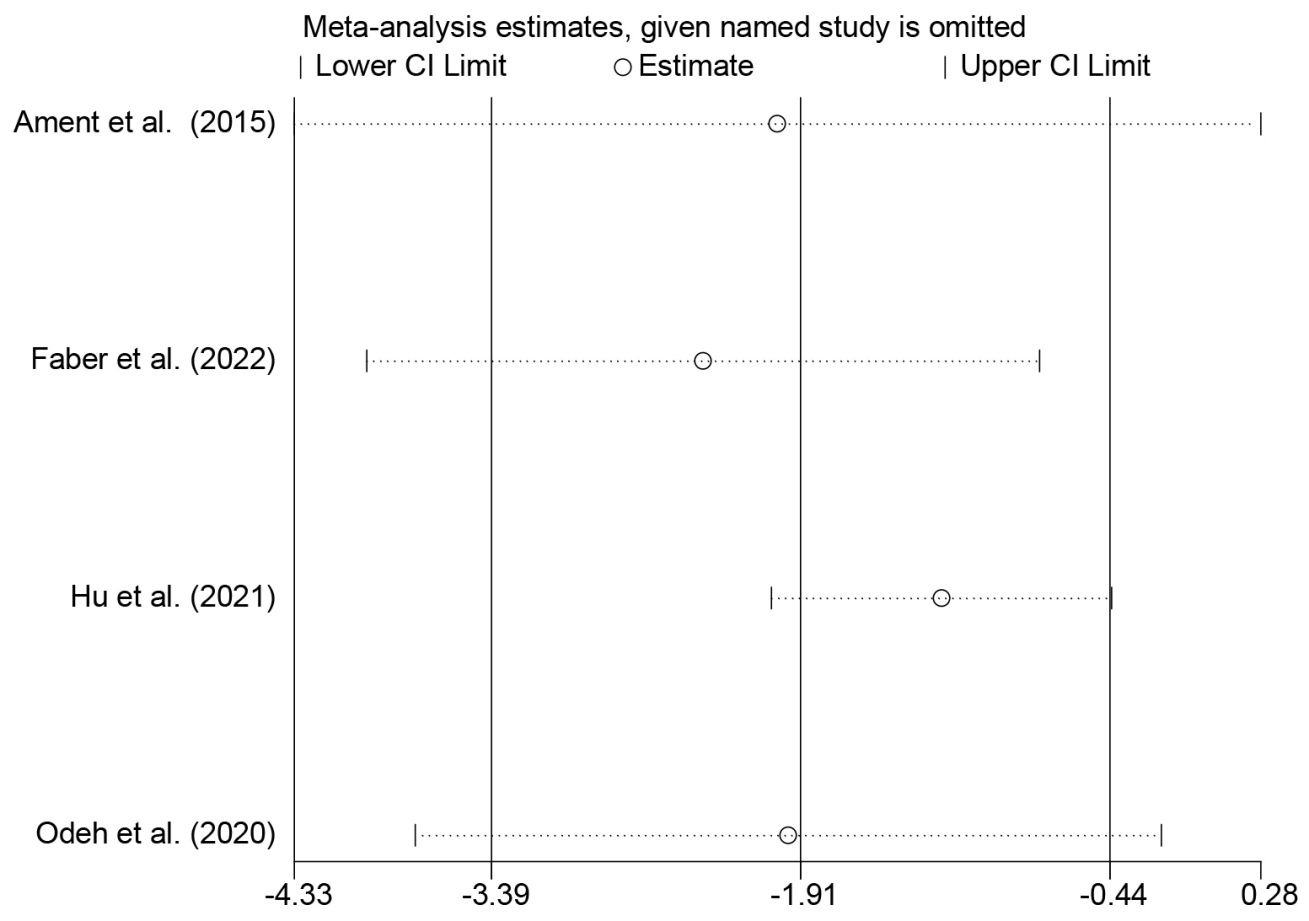
**The sensitivity analysis on the pooled results of the MABC-2 balance subscale**. MABC-2, Movement Assessment Battery for Children-2.

Due to the high heterogeneity, we performed a sensitivity analysis on the combined results of the MABC-2 balance subscale. The results showed that each study had no significant influence on the conclusion of the pooled effect of the primary outcome, suggesting that the robustness of outcome in the meta-analysis is robust (Fig. 4).

### 3.10 Publication Bias

**Fig. 5.**
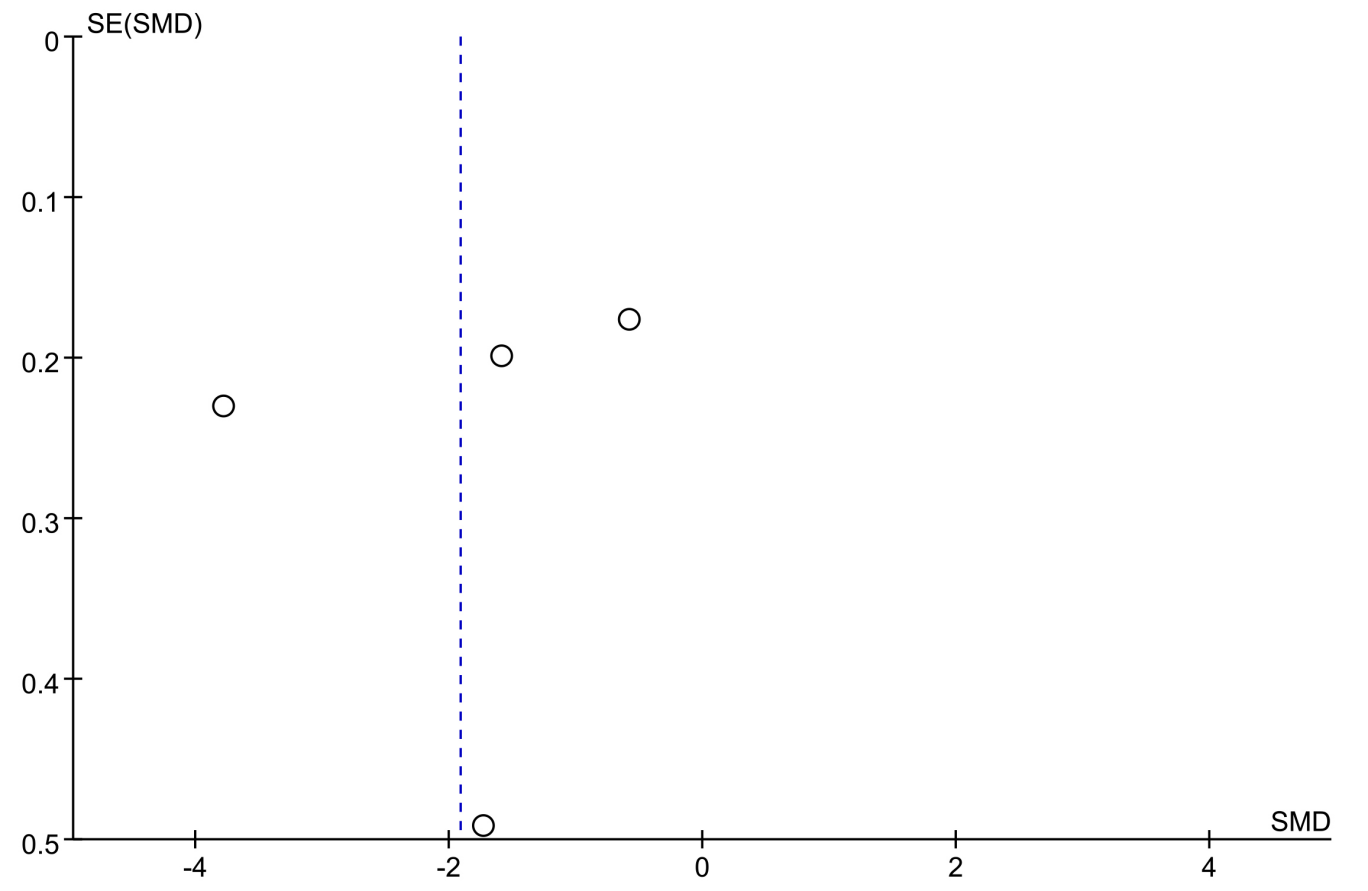
**The funnel plot of on the pooled results of the MABC-2 balance subscale**. SMD, standardised mean difference.

We further assessed the publication bias. The funnel plot showed poor symmetry in the distribution of scatter points (Fig. 5). However, the results of Egger’s test did not show a significant publication bias among the included studies (*t* = –0.45, *p* = 0.698).

## 4. Discussion

The purpose of this systematic review and meta-analysis was to explore various balance deficits in children and youth with ASD by comparing their balance performance with that of TD peers. It was found that individuals with ASD performed significantly more poorly in overall balance control than their TD peers (Fig. 2). In the various balance domains reviewed, children and youth with ASD encountered significantly more difficulties in the domain of sensory orientation (Fig. 3). They also experienced more hardship in the domains of verticality and anticipatory postural adjustments than their TD peers (**Supplementary File 2**).

This review found that children and youth with ASD had lower scores on the MABC-2 balance subscale than their TD peers (pooled SMD = –1.91, 95 % CI: –3.39 to –0.44). Moreover, results revealed significant heterogeneity (*I*^2^ = 98 %) amongst the studies analysed. This high heterogeneity may be due to the differences in sample characteristics across the included studies. Participants with a diagnosis of ASD were included on the basis of the inclusion criteria. However, a high proportion of children diagnosed with autistic disorder are also diagnosed with comorbidities, such as intellectual disabilities, attention-deficit hyperactivity disorder and depression. Therefore, participants with these comorbid conditions were also included in the present analyses [28, 29, 30, 39]. Additionally, differences in methodological quality of the included studies may also have been a source of heterogeneity. Of the combined evidence, only one study was rated as high quality, which may hinder the comparability of outcomes between the studies. The included age groups also differed among studies pooled in the meta-analyses. Given the small number of studies adopting the same outcome measures (i.e., the MABC-2 balance subscale), subgroup analysis (e.g., from the perspective of comorbidity, methodological quality or age) could not be performed. Hence, studies differences could not be explained accurately.

Since children with ASD exhibit deficient postural control in infancy [42], it is not surprising that they continue to present decreased balance control ability as they grow. The findings reported here are in line with that of previous reviews [43, 44], which reported that children with ASD had impairments in balance control skills. Unfortunately, even though parent perceptions of impaired motor development negatively impacts social cognition, communication and participation in children with ASD, the motor deficiencies associated with ASD do not induce many parents to seek treatment or a diagnosis [9].

In terms of various balance domains, the present meta-analysis showed that children and youth with ASD performed more poorly than their TD peers on tests of sensory orientations (the calculated effect sizes was 0.97), indicating large inter-group differences. This review found that during bipedal stance with eyes open, participants with ASD presented significantly larger COP sway than their TD peers. Sensory organisational processes are essential for balancing control, wherein multimodal sensory systems (e.g., somatosensory, visual and vestibular) are involved and integrated within the central nervous system [45]. Individuals with ASD have been found to exhibit impairment in the sensorimotor integration of visual, vestibular and somatosensory components at different levels of the central nervous system [37]. According to Ornitz *et al*. (1985) [46], the disorders to integrate sensory information result from the dysfunction of multisynaptic pathways in the brainstem. Evidence from functional magnetic resonance imaging suggests that sensory integration may occur at the cortical level. Specifically, visual and somatic sensory signals bilaterally converge in the posterior parietal and frontal cortices to create multimodal integrated spatial representations in a body-centred coordinate framework [47]. Moreover, the cerebellum may also be a site for the integration of sensory information [38]. Consistent with the finding of dysfunction in the integration of sensory input [38], neuroanatomical abnormalities in the parietal region and cerebellum have been described in neuroimaging studies of children with autism [48, 49].

Additionally, children with ASD have few opportunities to participate in physical activity and play with their peers due to impairments in social interaction and communication skills [50]. They therefore often present with low motivation to actively explore the environment and consequently experience few sensory inputs (e.g., visual and somatosensory inputs). This situation is likely to exacerbate difficulties in the function of sensory orientation.

Notably, amongst the ASD group without mental retardation, sensory integration problems were found to increase with the severity of autism disorder [37]. Given that few included studies assessed balance control ability on the basis of autism severity, further synthesising evidence to explore the influence of autism severity on sensory orientation problems is difficult. Additional studies are therefore needed to examine the influence of the differences in the severity of ASD on sensory orientation to design effective intervention programmes for this target population.

The results of this review also demonstrate that individuals with ASD have lower abilities in the domains of anticipatory postural adjustments and verticality than their TD peers. Decreased muscle strength and joint hypermobility may be the main causes for these impairments [6, 8]. However, due to the lack of studies discussing these balance areas, the current findings need further verification.

To the best of the authors’ knowledge, this review is the first systematic analysis to reveal balance deficits in children and youth with ASD in various balance domains. Its findings suggest further research directions and practical implications. Firstly, under the BESTest framework, detailed insights into the domains and extent of balance deficits present in children and youth with ASD would help health professionals, teachers and policy makers to develop targeted interventions to improve the balanced performance of this study population. Secondly, studies included in this review investigated some balance domains in ASD participants through the application of diverse tests or tasks. Further studies are warranted to comprehensively assess the entire construct of balance control within the ASD group. Finally, all of the studies included in this review applied a cross-sectional research design. Although cross-sectional research may allow generalization of findings to the whole group of ASD, additional longitudinal investigations are needed to explore the developmental process of balance control in this population.

## 5. Limitations

The limitations of this study include: (1) Although an extensive literature search was conducted to identify all published studies, a few published works possibly missed inclusion in this review due to their keywords not being captured by those used in the current work, along with vague titles or abstracts; (2) All included studies were cross-sectional, which may have involved selection bias; and (3) The exclusion of non-English published studies could have missed information relevant for this field.

## 6. Conclusions

This systematic review and meta-analysis highlights that children and youth with ASD have greater difficulties with balance control than their TD peers. Specifically, when compared to TD peers, individuals with ASD encountered greater difficulty in maintaining a stable standing position in sensory conditions. They also presented poorer abilities in the domains of anticipatory postural adjustments and verticality. To better design and implement more targeted intervention programs, further studies should fully assess the balance control construct within the ASD population, with longitudinal designs being encouraged.

## Data Availability

Data to support the findings of this study are available on reasonable request from the corresponding author.

## Supplementary Material

Supplementary material associated with this article can be found, in the online version, at https://doi.org/10.31083/AP42869.
