# Supplementary material for: Balance Control in Children and Youth with Autism Spectrum Disorder: A Systematic Review and Meta-Analysis
Source: Alpha Psychiatry. 2025 Jun 4;26(3):42869. doi: 10.31083/AP42869 (PMC12231408; doi:10.31083/AP42869)
Supplement: Supplementary file 1 [file 2757-8038-26-3-42869-s1.zip › Supplementary Files.doc]

**Supplementary File 1 - Search query and databases.**

| **Database** | **Search queries** |
| --- | --- |
| PubMed | ("Autism"[Title/Abstract] OR "autism spectrum disorder*"[Title/Abstract] OR "ASD"[Title/Abstract]) AND ("child*"[Title/Abstract] OR "adolescent*"[Title/Abstract] OR "youth*"[Title/Abstract] OR "pupil*"[Title/Abstract] OR "student*"[Title/Abstract] OR "teenager*"[Title/Abstract] OR "youngster*"[Title/Abstract]) AND ("postural balance"[Title/Abstract] OR "postural control"[Title/Abstract] OR "posturography"[Title/Abstract] OR "postural sway"[Title/Abstract] OR "posture*"[Title/Abstract] OR "balanc*"[Title/Abstract] OR "balanc* control"[Title/Abstract]) |
| Web of Science | ((TS=(Autism OR "autism spectrum disorder*" OR ASD)) AND TS=(child* OR adolescent* OR youth* OR pupil* OR student* OR teenager* OR youngster*)) AND TS=("postural balance" OR "postural control" OR posturography OR "postural sway" OR posture* OR balanc* OR "balanc* control") |
| Scopus | (TITLE-ABS-KEY("autism spectrum disorder") OR TITLE-ABS-KEY(Autism) OR TITLE-ABS-KEY(ASD)) AND (TITLE-ABS-KEY("postural balance") OR TITLE-ABS-KEY("postural control") OR TITLE-ABS-KEY(posturography OR posture OR balance) OR TITLE-ABS-KEY("postural sway")) AND (TITLE-ABS-KEY(child OR children) OR TITLE-ABS-KEY(teenager OR adolescent OR pupil OR youth OR young OR student)) |
| EBSCO | SU (Autism OR "autism spectrum disorder*" OR ASD) AND SU ("postural balance" OR "postural control" OR posturography OR "postural sway" OR posture* OR balanc* OR "balanc* control") AND SU (child* OR adolescent* OR youth* OR pupil* OR student* OR teenager* OR youngster*) |

**Supplementary File 2 - Raw data and SMD for performance of specific balance control tests in the included studies.**

| **Study** | **Tests/tasks** | **Outcome variables** | **Condition** | **ASD group** | **TD group** | **SMD (Hedges’g)** | | ***p*-value** |
| --- | --- | --- | --- | --- | --- | --- | --- | --- |
|  |  |  |  |  |  | **Mean** | **95% CI** |  |
| Kohen-Raz et al. 1992 [32] | Tetra-Ataxiametric Method | Stability index (no units) | EO | Age 7-9: 100.2 ± 56.4  Age 10-12: 131.5 ± 89.3  Age 13-20: 82.7 ± 58.7 | Age 5-7: 47 ± 16.6  Age 8-9: 34 ± 13.7  Age 10-11: 30 ± 12.2 | Not carried out | Not carried out | Not carried out |
|  |  |  | EC | Age 7-9: 93.2 ± 54.0  Age 10-12: 104.9 ± 48.5  Age 13-20: 84.6 ± 62.2 | Age 5-7: 52 ± 22.0  Age 8-9: 38 ± 11.7  Age 10-11: 36 ± 13.5 |  |  |  |
|  |  |  | PO | Age 7-9: 80.4 ± 43.0  Age 10-12: 97.0 ± 59.4  Age 13-20: 67.4 ± 26.2 | Age 5-7: 62 ± 20.3  Age 8-9: 44 ± 16.5  Age 10-11: 35 ± 10.1 |  |  |  |
|  |  |  | PC | Age 7-9: 88.1 ± 48.2  Age 10-12: 99.0 ± 31.1  Age 13-20: 87.0 ± 37.4 | Age 5-7: 87 ± 25.1  Age 8-9: 57 ± 19.6  Age 10-11: 53 ± 17.4 |  |  |  |
|  |  | Fourier spectral quotient  (no units) | EO | Age 7-9: 66.9 ± 18.6  Age 10-12: 60.8 ± 31.0  Age 13-20: 69.7 ± 22.6 | Age 5-7: 85 ± 37.1  Age 8-9: 88 ± 38.1  Age 10-11: 78 ± 28.9 | Not carried out | Not carried out | Not carried out |
|  |  |  | EC | Age 7-9: 72.3 ± 28.4  Age 10-12: 66.6 ± 27.9  Age 13-20: 79.8 ± 33.6 | Age 5-7: 83 ± 31.3  Age 8-9: 81 ± 30.7  Age 10-11: 76 ± 27.8 |  |  |  |
|  |  |  | PO | Age 7-9: 77.3 ± 30.4  Age 10-12: 62.2 ± 28.2  Age 13-20: 68.9 ± 26.8 | Age 5-7: 70 ± 24.7  Age 8-9: 78 ± 28.5  Age 10-11: 85 ± 29.3 |  |  |  |
|  |  |  | PC | Age 7-9: 69.1 ± 40.5  Age 10-12: 56.4 ± 22.8  Age 13-20: 59.8 ± 20.2 | Age 5-7: 59 ± 18.3  Age 8-9: 66 ± 23.8  Age 10-11: 88 ± 33.4 |  |  |  |
|  |  | Weight distribution index  (no units) | EO | Age 7-9: 9.3 ± 4.4  Age 10-12: 9.7 ± 4.2  Age 13-20: 7.3 ± 4.5 | Age 5-7: 6.3 ± 2.8  Age 8-9: 6.2 ± 2.9  Age 10-11: 6.4 ± 2.9 | Not carried out | Not carried out | Not carried out |
|  |  |  | EC | Age 7-9: 8.0 ± 6.2  Age 10-12: 8.2 ± 2.9  Age 13-20: 7.3 ± 3.8 | Age 5-7: 6.9 ± 3.0  Age 8-9: 6.1 ± 3.0  Age 10-11: 5.6 ± 3.1 |  |  |  |

**Supplementary** File 2 - Raw data and SMD for performance of specific balance control tests in the included studies - continued.

| **Study** | **Tests/tasks** | **Outcome variables** | **Condition** | **ASD group** | **TD group** | **SMD (Hedges’g)** | | ***p*-value** |
| --- | --- | --- | --- | --- | --- | --- | --- | --- |
|  |  |  |  |  |  | **Mean** | **95% CI** |  |
| Kohen-Raz et al. 1992 [32] | Tetra-  Ataxiametric Method | Weight distribution index (no units) | PO | Age 7-9: 7.7 ± 5.0  Age 10-12: 10.0 ± 5.1  Age 13-20: 7.6 ± 3.7 | Age 5-7: 5.7 ± 2.7  Age 8-9: 6.0 ± 3.3  Age 10-11: 6.0 ± 3.1 | Not carried out | Not carried out | Not carried out |
|  |  |  | PC | Age 7-9: 8.5 ± 6.1  Age 10-12: 9.2 ± 4.9  Age 13-20: 7.8 ± 3.8 | Age 5-7: 6.1 ± 3.0  Age 8-9: 5.6 ± 2.8  Age 10-11: 5.6 ± 3.0 |  |  |  |
|  |  | Toe synchronization  (no units) | EO | Age 7-9: 2.2 ± 46.5  Age 10-12: -14.2 ± 54.3  Age 13-20: 4.9 ± 42.8 | Age 5-7: 40 ± 32.5  Age 8-9: 44 ± 30.3  Age 10-11: 43 ± 36.0 | Not carried out | Not carried out | Not carried out |
|  |  |  | EC | Age 7-9: 28.5 ± 44.2  Age 10-12: 22.4 ± 56.0  Age 13-20: 22.3 ± 42.0 | Age 5-7: 56 ± 27.4  Age 8-9: 53 ± 29.9  Age 10-11: 53 ± 28.2 |  |  |  |
|  |  |  | PO | Age 7-9: 18.7 ± 44.7  Age 10-12: -10.1 ± 61.2  Age 13-20: -3.4 ± 36.5 | Age 5-7: 21 ± 38.2  Age 8-9: 31 ± 39.7  Age 10-11: 24 ± 41.9 |  |  |  |
|  |  |  | PC | Age 7-9: 14.7 ± 59.2  Age 10-12: 2.5 ± 52.0  Age 13-20: -0.1 ± 47.4 | Age 5-7: 43 ± 31.7  Age 8-9: 38 ± 30.6  Age 10-11: 30 ± 39.1 |  |  |  |
| Molloy et al. 2003 [38] | Force platform-bipedal stance | COP-area (cm^2^) | EO | 4.70 | 3.30 | Not carried out | Not carried out | Not carried out |
|  |  |  | EC | 8.67 | 4.48 |  |  |  |
|  |  |  | PO | 7.99 | 5.40 |  |  |  |
|  |  |  | PC | 14.10 | 8.60 |  |  |  |
| Minshew et al. 2004 [37] | EquiTest | Equilibrium score  (no units) | EO | **88.2 ± 7.4** | **90.9 ± 5.0** | **-0.42** | **[-0.75, -0.08]** | **0.02** |
|  |  |  | EC | **84.4 ± 8.0** | **87.1 ± 5.9** | **-0.37** | **[-0.71, -0.04]** | **0.03** |
|  |  |  | EO/Sway-referenced support surface | **72.0 ± 15.6** | **77.4 ± 14.9** | **-0.35** | **[-0.69, -0.01]** | **0.04** |
|  |  |  | EC/Sway- referenced support surface | **51.4 ± 20.3** | **59.4 ± 15.7** | **-0.43** | **[-0.77, -0.09]** | **0.01** |

**Supplementary** File 2 - Raw data and SMD for performance of specific balance control tests in the included studies - continued.

| **Study** | **Tests/tasks** | **Outcome variables** | **Condition** | **ASD group** | **TD group** | **SMD (Hedges’g)** | | ***p*-value** |
| --- | --- | --- | --- | --- | --- | --- | --- | --- |
|  |  |  |  |  |  | **Mean** | **95% CI** |  |
| Fournier et al. 2010 [30] | Force Plates-bipedal stance | COP-ML (no units) | EO | 0.388 ± 0.567 | 0.072 ± 0.033 | 0.74 | [-0.07, 1.56] | 0.07 |
|  |  | COP-AP (no units) | EO | **0.192 ± 0.076** | **0.094 ± 0.043** | **1.52** | **[0.61, 2.43]** | **0.001** |
|  |  | COP-area (no units) | EO | 0.104 ± 0.159 | 0.007 ± 0.005 | 0.82 | [-0.01, 1.64] | 0.05 |
|  |  | COP-COM _max_ML_  (no units) | EO | 0.029 ± 0.032 | 0.013 ± 0.008 | 0.65 | [-0.16, 1.46] | 0.11 |
|  |  | COP-COM _max_AP_  (no units) | EO | **0.016 ± 0.010** | **0.008 ± 0.003** | **1.03** | **[0.19, 1.87]** | **0.02** |
|  |  | COP-COM _max_R_  (no units) | EO | **0.037 ± 0.033** | **0.015 ± 0.008** | **0.87** | **[0.04, 1.70]** | **0.04** |
| Liu & Breslin, 2013 [35] | MABC-2 (balance subtests) | Percentiles score |  | NR | NR |  |  |  |
| Memari et al. 2013 [36] | Force Plates-bipedal stance | COP-AP (cm) | EO | **3.47 ± 1.68** | **1.74 ± 0.69** | **1.42** | **[0.80, 2.05]** | **< 0.001** |
|  |  | COP-ML (cm) | EO | **4.82 ± 4.55** | **1.27 ± 0.68** | **1.18** | **[0.58, 1.79]** | **< 0.001** |
|  |  | COP-RMS-AP (cm) | EO | **0.66 ± 0.33** | **0.33 ± 0.15** | **1.35** | **[0.73, 1.97]** | **< 0.001** |
|  |  | COP-RMS-ML (cm) | EO | **0.87 ± 0.89** | **0.22 ± 0.13** | **1.11** | **[0.51, 1.71]** | **< 0.001** |
|  |  | COP-RMS (cm) | EO | **1.15 ± 0.89** | **0.41 ± 0.18** | **1.25** | **[0.63, 1.86]** | **< 0.001** |
|  |  | COP-AP-V (cm/s) | EO | 4.28 ± 2.28 | 3.56 ± 2.45 | 0.30 | [-0.26, 0.86] | 0.30 |
|  |  | COP-ML-V (cm/s) | EO | **7.43 ± 5.82** | **4.44 ± 1.54** | **0.75** | **[0.18, 1.33]** | **0.01** |
|  |  | COP-V (cm/s) | EO | **9.49 ± 5.83** | **6.34 ± 2.88** | **0.72** | **[0.14, 1.29]** | **0.01** |
|  |  | COP-area (cm^2^/s) | EO | **63.82 ± 73.61** | **15.66 ± 9.87** | **1.00** | **[0.40, 1.59]** | **0.001** |
|  |  | MF (Hz) | EO | **2.04 ± 1.23** | **3.21 ± 1.75** | **-0.74** | **[-1.32, -0.16]** | **0.01** |
| Fournier et al. 2014 [31] | Force platform-bipedal stance | COP-AP (cm) | EO | 5.89 ± 3.37 | 4.01 ± 1.39 | 0.72 | [0.01, 1.43] | 0.05 |
|  |  | COP-ML (cm) | EO | **6.51 ± 4.92** | **3.33 ± 0.96** | **0.89** | **[0.17, 1.61]** | **0.02** |
|  |  | COP-V (cm/s) | EO | **4.04 ± 3.83** | **1.87 ± 0.62** | **0.78** | **[0.07, 1.50]** | **0.03** |
|  |  | COP-area _CE95_ (cm^2^) | EO | 67.76 ± 119.99 | 6.88 ± 5.38 | 0.71 | [0.00, 1.42] | 0.05 |
| Ament et al. 2015 [28] | MABC-2 (balance subtests) | Standard score |  | **4.82 ± 2.59** | **9.27 ± 2.92** | **-1.59** | **[-1.98, -1.20]** | **< 0.001** |

**Supplementary** File 2 - Raw data and SMD for performance of specific balance control tests in the included studies - continued.

| **Study** | **Tests/tasks** | **Outcome variables** | **Condition** | **ASD group** | **TD group** | **SMD (Hedges’g)** | | ***p*-value** |
| --- | --- | --- | --- | --- | --- | --- | --- | --- |
|  |  |  |  |  |  | **Mean** | **95% CI** |  |
| Wang et al. 2016 [39] | Force Plates-bipedal stance | COP-AP-standard deviation (cm) | EO/Static stance | NR | NR |  |  |  |
|  |  |  | EO/AP-sway | NR | NR |  |  |  |
|  |  |  | EO/ML-sway | NR | NR |  |  |  |
|  |  | COP-ML-standard deviation (cm) | EO/Static stance | NR | NR |  |  |  |
|  |  |  | EO/AP-sway | NR | NR |  |  |  |
|  |  |  | EO/ML-sway | NR | NR |  |  |  |
|  |  | COP-length (cm) | EO/Static stance | NR | NR |  |  |  |
|  |  |  | EO/AP-sway | NR | NR |  |  |  |
|  |  |  | EO/ML-sway | NR | NR |  |  |  |
| Lim et al. 2020 [34] | Force Plates-bipedal stance | COP-AP (mm) | EO | 3.41 ± 12.53 | -0.60 ± 6.90 | 0.40 | [-0.30, 1.09] | 0.26 |
|  |  |  | EC | 1.89 ± 9.76 | -0.12 ± 5.10 | 0.26 | [-0.43, 0.95] | 0.46 |
|  |  | COP-ML (mm) | EO | 0.31 ± 7.45 | -2.02 ± 5.02 | 0.36 | [-0.33, 1.06] | 0.30 |
|  |  |  | EC | -0.78 ± 5.83 | -2.24 ± 4.95 | 0.27 | [-0.42, 0.95] | 0.45 |
|  |  | COP-RMS-AP (mm) | EO | 7.43 ± 3.35 | 6.21 ± 3.46 | 0.35 | [-0.34. 1.04] | 0.32 |
|  |  |  | EC | 8.10 ± 3.53 | 7.00 ± 3.78 | 0.29 | [-0.40, 0.98] | 0.41 |
|  |  | COP-RMS-ML (mm) | EO | 5.47 ± 3.77 | 3.89 ± 1.26 | 0.57 | [-0.13, 1.27] | 0.11 |
|  |  |  | EC | 5.05 ± 1.40 | 3.91 ± 1.64 | 0.72 | [0.01, 1.43] | 0.05 |
|  |  | COP-V (mm/s) | EO | 19.97 ± 10.98 | 16.69 ± 4.86 | 0.39 | [-0.30, 1.08] | 0.27 |
|  |  |  | EC | 23.37 ± 11.66 | 18.86 ± 5.30 | 0.50 | [-0.20, 1.20] | 0.16 |
|  |  | SE_-AP_ | EO | 1.14 ± 0.20 | 1.20 ± 0.19 | -0.30 | [-0.99, 0.39] | 0.39 |
|  |  |  | EC | 1.20 ± 0.20 | 1.23 ± 0.18 | -0.15 | [-0.84, 0.53] | 0.66 |
|  |  | SE_-ML_ | EO | 1.09 ± 0.19 | 1.11 ± 0.17 | -0.11 | [-0.79, 0.58] | 0.76 |
|  |  |  | EC | 1.13 ± 0.22 | 1.13 ± 0.17 | 0.00 | [-0.69, 0.69] | 1.00 |
| Lidstone et al. 2020 [33] | Force Plates-bipedal stance | COP-area _CE95_ (cm^2^) | EO | NR | NR |  |  |  |
|  |  | COP-RMS-ML (cm) | EO | NR | NR |  |  |  |
|  |  | COP-RMS-AP (cm) | EO | NR | NR |  |  |  |

**Supplementary** File 2 - Raw data and SMD for performance of specific balance control tests in the included studies - continued.

| **Study** | **Tests/tasks** | **Outcome variables** | **Condition** | **ASD group** | **TD group** | **SMD (Hedges’g)** | | ***p*-value** |
| --- | --- | --- | --- | --- | --- | --- | --- | --- |
|  |  |  |  |  |  | **Mean** | **95% CI** |  |
| Lidstone et al. 2020 [33] | Force Plates-bipedal stance | COP-ML-V (cm/s) | EO | NR | NR |  |  |  |
|  |  | COP-AP-V (cm/s) | EO | NR | NR |  |  |  |
|  |  | SE_-ML_ | EO | NR | NR |  |  |  |
|  |  | SE_-AP_ | EO | NR | NR |  |  |  |
|  | Unipedal stance test | Unipedal stance time (s) | EO | NR | NR |  |  |  |
| Hu et al. 2021 [26] | MABC-2 (balance subtests) | Standard score |  | **1.64 ± 1.75** | **9.77 ± 2.43** | **-3.77** | **[-4.22, -3.32]** | **< 0.001** |
| Odeh et al. 2020 [9] | BOT-2  (balance subtest) | Standard scores |  | **9.090 ± 2.700** | **14.000 ± 3.411** | **-1.54** | **[-2.47, -0.61]** | **0.001** |
|  | MABC-2 (balance subtest) | Standard score |  | **5.640 ± 1.859** | **9.830 ± 2.758** | **-1.72** | **[-2.68, -0.76]** | **< 0.001** |
| Faber et al. 2022 [29] | MABC-2 (balance subtests) | Standard score |  | **8.20 ± 2.85** | **9.66 ± 2.21** | **-0.57** | **[-0.91, -0.22]** | **0.001** |
| Abdel Ghafar et al. 2022 [27] | Biodex balance system | Postural sway index | EO/Firm surface | **5.73 ± 1.34**  **3.26 ± 1.15** | **0.71 ± 0.07**  **0.59 ± 0.03** | **5.16**  **3.20** | **[4.20, 6.13]**  **[2.50, 3.90]** | **< 0.001**  **< 0.001** |
|  |  |  | EC/Firm surface | **5.24 ± 1.71**  **4.47 ± 1.72** | **1.69 ± 0.09**  **1.07 ± 0.08** | **2.86**  **2.73** | **[2.20, 3.52]**  **[2.08, 3.37]** | **< 0.001**  **< 0.001** |
|  |  |  | EO/Foam surface | **5.06 ± 2.21**  **3.16 ± 0.87** | **1.34 ± 0.02**  **0.97 ± 0.3** | **2.32**  **3.29** | **[1.73, 2.92]**  **[2.58, 4.01]** | **< 0.001**  **< 0.001** |
|  |  |  | EC/Foam surface | **7.21 ± 1.68**  **6.78 ± 2.11** | **2.02 ± 0.26**  **1.45 ± 0.13** | **4.22**  **3.48** | **[3.38, 5.05]**  **[2.75, 4.22]** | **< 0.001**  **< 0.001** |
|  |  | Postural sway score | Overall sway index score | **6.58 ± 0.62**  **4.25 ± 1.15** | **1.50 ± 0.04**  **1.03 ± 0.07** | **11.29**  **3.86** | **[9.36, 13.21]**  **[3.07, 4.64]** | **< 0.001**  **< 0.001** |

**Supplementary** File 2 - Raw data and SMD for performance of specific balance control tests in the included studies - continued.

| **Study** | **Tests/tasks** | **Outcome variables** | **Condition** | **ASD group** | **TD group** | **SMD (Hedges’g)** | | ***p*-value** |
| --- | --- | --- | --- | --- | --- | --- | --- | --- |
|  |  |  |  |  |  | **Mean** | **95% CI** |  |
| Stania et al. 2023 [18] | Force Plates-bipedal stance | COP-AP (cm) | EO | NR | NR |  |  |  |
|  |  | COP-ML (cm) | EO | NR | NR |  |  |  |
|  |  | COP-AP rambling (cm) | EO | NR | NR |  |  |  |
|  |  | COP-ML rambling (cm) | EO | NR | NR |  |  |  |
|  |  | COP-AP trembling (cm) | EO | NR | NR |  |  |  |
|  |  | COP-ML trembling (cm) | EO | NR | NR |  |  |  |
|  |  | COP-RMS-AP (cm) | EO | NR | NR |  |  |  |
|  |  | COP-RMS-ML (cm) | EO | NR | NR |  |  |  |
|  |  | COP-RMS-AP rambling (cm) | EO | NR | NR |  |  |  |
|  |  | COP-RMS-ML rambling (cm) | EO | NR | NR |  |  |  |
|  |  | COP-RMS-AP trembling (cm) | EO | NR | NR |  |  |  |
|  |  | COP-RMS-ML trembling (cm) | EO | NR | NR |  |  |  |
|  |  | SE_-AP_ | EO | NR | NR |  |  |  |
|  |  | SE_-ML_ | EO | NR | NR |  |  |  |

Note: bold values indicate the 95% CI does not include zero.

The omission of SMD calculations was necessitated by (a) inconsistent age-group aggregation in reported data (Kohen-Raz et al., 1992), (b) not fulfil the conditions for carrying out the calculation (Molloy et al., 2003), and (c) reliance on graphical summaries without raw numerical values in multiple studies (Liu & Breslin, 2013; Lidstone et al., 2020; Stania et al., 2023; Wang et al., 2016).

Abbreviations: ASD: autism spectrum disorder; TD: typically developing; SMD: standardized mean differences; CI: confidence interval; EO: eyes open; EC: eyes closed; PO: eyes open, standing on pads; PC: eyes closed, standing on pads; COP: centre of pressure; COM: centre of mass; AP: anterior-posterior; ML: medial-lateral; V: velocity; RMS: root mean square; COP-area _CE95_: elliptical sway area containing 95% of the COP data; SE: sample entropy; MF: Mean frequency; BOT-2: Bruininks Oseretsky Test of Motor Proficiency, Second Edition; MABC-2: Movement Assessment Battery for Children, Second Edition; NR: not reported.
